# A new self-understanding as chemo sufferer - a phenomenological study of everyday life with chemotherapy induced neuropathy among survivors after colorectal cancer

**DOI:** 10.1080/17482631.2022.2049437

**Published:** 2022-03-22

**Authors:** Marlene AE. Jensen, Carsten D. Mørch, Mette N. Yilmaz, Casper Feilberg, Birgith Pedersen

**Affiliations:** a Department of Oncology, Clinic for Surgery and Cancer treatment, Aalborg University Hospital, Denmark; b Department of Health Science and Technology, Aalborg University, Aalborg, Denmark; c Department of Oncology, Clinic for Surgery and Cancer treatment, Aalborg University Hospital, Denmark; Clinical Cancer Research Centre, Aalborg University Hospital, Denmark; d Department of Communication and Psychology, Aalborg University, Aalborg, Denmark; e Department of Oncology, Clinic for Surgery and Cancer treatment, Aalborg University Hospital, Denmark; Clinical Nursing Research Unit, Aalborg University Hospital, Denmark; Clinical Cancer Research Centre, Aalborg University Hospital, Aalborg, Denmark

**Keywords:** Colorectal cancer, peripheral neuropathy, long-term side effects, cancer survivorship, descriptive life-world research, embodiment, existential phenomenology, quality of life, everyday life

## Abstract

**Purpose:**

To explore the essential meaning of how sensory disturbances caused by Oxaliplatin influence self-understanding and freedom to live an everyday life among survivors after colorectal cancer.

**Methods:**

Data was generated by means of a semi-structured individual interview with eight survivors after colorectal cancer who continued to experience chemotherapy-induced peripheral neuropathy at least one year after completing chemotherapy with Oxaliplatin. Data analysis was guided by existential phenomenology and descriptive life-world research.

**Results:**

The essential meaning was structured by four constituents. 1) An unpleasant fluctuating sensation which is impossible to ignore, 2) Breaking through of noise and pain despite struggling to keep them at bay, 3) Continuously feeling ill despite being cured, and 4) Bodily constraints that impact self-understanding and limit enjoyment of life.

**Conclusion:**

The survivors used distraction to keep the sensory disturbances at bay but were forced to adapt to a new self-understanding as sufferers after chemotherapy despite being cured of their cancer disease. This way of being-in-the-world was understood by survivors, their families and healthcare professionals as a necessary price to pay to be alive. However, marked as sufferer after chemotherapy, the participants’ everyday style of experience and life revealed as an ill health condition, which limited their ability to accomplish everyday activities as before and their freedom to realize their potential—the “I can”.

## Introduction

As a result of improved surgery, chemotherapy and screening programs for colorectal cancer (CRC), overall survival has improved significantly (Danish Colorectal Cancer Group, 2020). However, after receiving adjuvant treatment with the chemotherapeutic drug Oxaliplatin, patients may suffer from long-term chemotherapy-induced neuropathy (CIPN). Although it is well known that these sensory disturbances influence survivors extensively and long-term, we lack knowledge about how this influences the survivors’ self-understanding and their freedom to conduct their everyday life.

## Background

In order to be cured, patients diagnosed with CRC at an early stage undergo surgery, followed by adjuvant chemotherapy regimens including the chemotherapeutic drug Oxaliplatin. Oxaliplatin is a platinum-based drug that contributes to efficient treatment with increased survival rates. However, this chemotherapeutic agent has been demonstrated to cause CIPN with an incidence rate from 70% up to 100% (Zajączkowska et al., 2019). CIPN affects the nervous system and may lead to sensory symptoms such as pain, tingling and burning and motor symptoms which can affect coordination, balance, walking, eating, and writing (Massey et al., 2014; Zajączkowska et al., 2019). Despite the treatment being administered with close monitoring to decrease the risk for persistent CIPN, it can vary in duration and intensity, emerge rapidly, develop, and increase months to years after treatment cessation (Kerckhove et al., 2017). In addition, research shows that CIPN is still present in 84% of patients at two years follow-up (Zajączkowska et al., 2019). No treatment has been found to eliminate these side effects and CIPN remains a challenge for survivors as well as health care providers (Zajączkowska et al., 2019).

Some of the challenges are described in studies that explore the experiences of CIPN and their influence on everyday living. In their study of daily living with CIPN, Kanda et al. (2017) identified several emotional challenges such as feelings of fear, helplessness, dismay and other uncomfortable feelings. In addition, they found an inability to perform daily activities and fear of being unable to live the same life as before (Kanda Fujimoto, & Kyota,, 2017). These findings supported Tofthagen (2010), who demonstrated how CIPN affected emotional and physical well-being and caused significant personal loss as the relationship with friends, family and co-workers was adversely changed due to functional limitations and inability to participate in usual activities. Another study showed how the influence on the survivors’ life situation and everyday life after treatment completion required the survivors to live with the distressing changes although they tried to cope by ignoring them (Drott et al., 2016). Recently, a study explored how survivors experience and give meaning to the embodied phenomenon of CIPN in everyday life after Oxaliplatin treatment for colorectal cancer. In this study CIPN was described as a peculiar feeling in the hands and feet that were difficult to verbalize and furthermore led to an alienation of the body and affected the ability to be in touch with the world and other people (Pedersen, Jensen, Yilmaz, Mørch, Feilberg , 2021).

Following these findings, being forced to tolerate a changed body seemed to affect self-understanding and agency as well as social relationships. Additionally, the sensory disturbances caused by CIPN seem to have serious consequences on the physical, psychological, and social aspects of the survivors’ everyday lives. Although the above studies showed that the disturbances influenced the survivors in several ways, none explored how the perceived embodied changes affected the survivors’ self-understanding that develops through lived experiences and therefore changes if one’s experience of everyday life and the world changes (Bell & Leite, 2016). Neither of the studies explored how the changes influenced the freedom to live everyday life despite the fact that a sense of freedom is situated and set by one’s bodily capacities, one’s history and the social contexts with other people (Compton, 1997). In the light of the above gap in the research literature, this study aims to explore the essential meaning of how sensory disturbances caused by Oxaliplatin influence self-understanding and freedom to live an everyday life among survivors after colorectal cancer.

## Design and method

### Philosophical perspective

To explore the experiences of sensory disturbances on self-understanding and freedom in everyday life, the study is designed and guided by existential phenomenology with its focus on perception and the embodied character of experiences (Compton, 1997). From this perspective, the physical body is an aspect of the embodied self as the human being is viewed as a unified inseparable body-subject (Merleau-Ponty, 2014). Through our embodied senses, language, emotion and movement in time and space, the embodied self is always directed against something and formed in inter-relatedness with the world, culture, and environment that all serves as a horizon or a background from which “something” stands out as significant figures, the gestalt (Merleau-Ponty, 2004, 2014). The body orients and directs itself to what it knows, remembers and understands in a sense other than the intellect (Moya, 2014). Therefore, an active embodied relationship between human consciousness, knowledge and the world, self-understanding or personal identity is bound to the living body through its function as a mediator to the world (Northoff, 1997). Thus, it is the “doing” or the “I can”, a bodily capacity that becomes essential. However, the “I can” depends on the person’s embodied sensorimotor and perceptual capacities that limit and empower the possibility for action in addition to the inter-relatedness with the world, culture, and environment. Moreover, self-understanding is related to intersubjectivity (relationships with others). Entering into relationships with others means that sickness and disabilities are recognized by seeing oneself in the eyes of other (Merleau-Ponty, 2014).

The freedom to act is the living body’s ability to realize its potential that is generated through the interaction between embodied subjects, the situation and interaction with others (Bakare-Yusuf, 2008). In addition, the ability to come into a new being is possible due to the “body scheme” that is an integrated part of the self and refers to a pre-conscious system of bodily movements and situational spatiality—our habits (Merleau-Ponty, 2014). Habits are not only mechanical processes but forms of knowledge that spare us from thinking. However, when the “I can” is limited, a discrepancy appears between the perception of a habitual body that normally acts flexibly and spontaneously and the actual body where movements require an intellectual reflection (Moya, 2014). Therefore, learning new habits requires a new use of the body and to reorganize the body scheme (Merleau-Ponty, 2014).

### Methodology

The analysis was guided by existential phenomenology (Merleau-Ponty, 2014) and draws methodologically on reflective life-world research, which is a way of analysing empirical data that emphasizes a bridled openness to the studied phenomenon (Dahlberg & Dahlberg, 2019). Following existential phenomenological methodology, phenomenological research is descriptive (Dahlberg & Dahlberg, 2019; Feilberg et al., 2018). It also includes an interpretive movement laying out the empirical material in a way that is as open and true to the phenomenon as possible and a secondary interpretation in the light of theory and other research findings in an attempt to understand more (Dahlberg & Dahlberg, 2019). From this approach, we aimed to illustrate survivors’ self-understanding and freedom to live their everyday life with sensory disturbances through a descriptive research method that was suitable to describe and elucidate the world we have access to through our bodies (Dahlberg et al., 2008). The manuscript is presented following the consolidated criteria for reporting qualitative research (Tong et al., 2007).

### Participants

In qualitative and phenomenological research, it is recommended to select participants who have experiences related to the studied phenomenon (Polit & Beck, 2012). In addition, it is anticipated that two to ten (Groenewald, 2004) or 15 ± 10 (Brinkmann & Kvale, 2015) participants are sufficient to reach saturation as variation is more important than numbers (Polit & Beck, 2012). Thus, we included eight participants purposefully (Polit & Beck, 2012); (Table I). Inclusion criteria were persons who experienced sensory disturbances at least one year after completing adjuvant treatment, were able to read and understand Danish and were willing to articulate their experience of everyday life with CIPN. Exclusion criteria were potential participants with pre-existing neuropathy e.g., related to diabetes or neurological diseases or cancer treatment with nerve toxic drugs other than oxaliplatin.

**Table I.** Characteristics of participants

Name	Age	Gender	Social status	Working status	Cancer type	Time since treatment cessation (time between interview and treatment)
Dot	76	Female	Living alone	Retired (due to age)	Colon	5 years and 10 months
Jack	67	Male	Living with wife	Retired (due to age)	Rectum	1 year and 3 months
Jean	72	Female	Living alone	Retired (due to age)	Colon	4 years and 6 months
Mary	57	Female	Living alone	Working part time (due to CIPN)	Colon	2 years and 7 months
Karen	63	Female	Living with husband	Working full time	Colon	11 years and 6 months
John	57	Male	Living with wife	Early retirement (due to CIPN)	Rectum	7 years
Robert	69	Male	Living with wife	Retired (due to age)	Colon	3 years and 9 months
Carl	62	Male	Living with wife	Early retirement (due to CIPN)	Rectum	6 years and 3 months

No relationship between the research group and participants was established before inclusion. In collaboration with the chairman from the local branch of the Danish Cancer Society, we searched for informants on a website aimed at survivors with late complications after colorectal cancer. The call contained brief information about the study and contact details of the research group. In total 11 persons responded but seven of these either did not meet the inclusion criteria or withdrew after the first telephone contact due to other illnesses. Later, clinical nurses consecutively recruited four eligible survivors when they visited the oncological outpatient clinic. None of the approached declined to participate.

#### Data gathering

A semi-structured interview guide focusing on lifeworld experiences of sensory disturbances supported the data gathering. The interview guide explored the experience of self-understanding, relation to others and how the participants were able to plan and accomplish activities that were meaningful for their lives (Bakitas, 2007; Dahlberg et al., 2008; Mohrmann, 2017); (Table II). The interview took place in a dialogue to allow the participants to describe their experiences. Following the participants’ preferences, five were interviewed at their homes and three at the hospital in an available office. The last author (a registered nurse with a PhD), without daily contact with patients, conducted all the interviews between February and May 2019. Data gathering continued until the aim was exhaustedly unfolded to gain a rich description of the phenomenon. The interviews lasted from 24 minutes to 64 minutes (mean 38). The recorded time does not include the introduction and establishing of the relationship between the participant and the interviewer nor the debriefing. If essential topics appeared in the introduction, questions about them were asked in the interviews and if the debriefing revealed something new, it was noted by the interviewer afterwards.

**Table II.** Interview guide

In your own words, how will you describe a regular day with sensory disturbances? Tell me about any of the ways having sensory disturbances have changed your life; e.g. how you move, leisure activities, working condition. Describe the meaning of and the context of these changes.. Tell me about any of the ways sensory disturbances have influenced the way you perceive yourself and describe some examples of how these appear for you. Tell me about how sensory disturbances have influenced your relationships with spouses/children, family, friends, colleagues, and what self-care activities you have used to relieve the neuropathic symptoms.

### Ethics

The project was conducted in accordance with the research ethics set out by The Northern Nurses Federation (Northern Nurses Federation, 2003) and the Helsinki Declaration (The World Medical Association, 2018). The participants were informed orally and in writing that their participation was voluntary and their right to withdraw from the project at any time without consequences before they signed an informed consent. In addition, the project was notified to the Danish Data Protection Agency (journal number (ID 2018 150) and data were stored in a safe place only accessible to the researchers. Anonymity was ensured by using pseudonyms for the participants’ names.

### Analysis

The aim of our analysis was to describe and understand the experiences of our participants as close to their lived, pre-reflective experience as possible, what phenomenology describes as returning to the matter itself (die Sachen selbst; Feilberg et al., 2018; Merleau-Ponty, 2004). First, the interviews were transcribed verbatim by a student after which the interviewer verified the transcript. Next, four transcriptions were read and analysed by all members of the research group. The group consisted of two registered nurses experienced in oncology with, respectively, a master’s degree in clinical nursing and a PhD in clinical nursing. In addition, a medical doctor, the team leader for the colorectal staff, a psychologist, and a biophysicist, who were employed at a university as associate professors, participated in the group. One by one, the researchers noted their immediate understanding of the phenomenon while staying open to the phenomenon (Dahlberg & Dahlberg, 2019; Zahavi, 2019). Next, there followed a systematic organization of all the empirical material inspired by Dahlberg et al. (2008) and their distinction between meaning units, clusters, constituents, and essential meaning. The meaning units that seemed to belong together were clustered in patterns that describe both essential meanings and the further constituents. Also, particularities and nuances that belong to the phenomenon were described (Dahlberg et al., 2008). See, Table III for an extract of this process. The essential meaning of a phenomenon (Dahlberg, 2006) denotes that which is stable within a phenomenon or an experience and is made up of, “essential qualities of elementary meaning and experience” (Feilberg et al., 2018, p. 222).

**Table III.** Extract of structuring data into clusters, constituents, and essential meaning

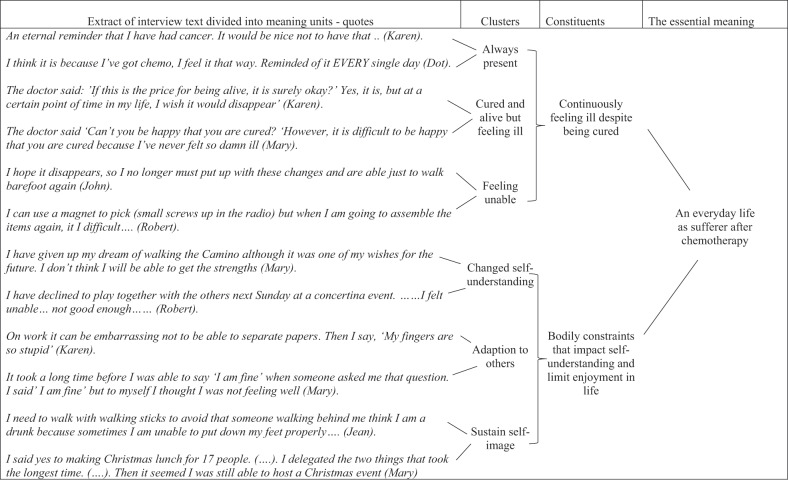

During the analysis and the reporting of findings, we applied the double movements of analysis considering the importance of including description and interpretation as part of phenomenological analysis (Dahlberg & Dahlberg, 2019; Feilberg et al., 2018). We moved between the empirical material, which included summaries of the participants descriptions (see, Table IV for an example), the investigated phenomenon and the presented phenomenological philosophy (Dahlberg & Dahlberg, 2019). Thus, we conducted the analysis in interplay between whole and parts. To stay close to the matter itself, the transcripts were analysed from a phenomenological attitude where taken for granted assumptions were bridled (Dahlberg, 2006). This helped us to restrain our pre-understandings such as personal beliefs and theories in order not to understand too quickly and too carelessly (Dahlberg, 2006).

**Table IV.** Mary’s story

Mary is 57 years old, living alone and she has sensory disturbances 2,5 years after completion of her cancer treatment. She has increasing pain in the joints and sensory disturbances (numbness and buzzing in the limps) that prevent her from sleeping. Hence, she suffers from tiredness/fatigue, which makes it difficult for her to perform physical activities. Her overwhelming fatigue limits her social life and impacts her self-understanding as one who often invites family and friends and who takes care of family and friends. She tries to compensate by preparing food over several days when she invites guests. Then she can hide her weakness. When she shares her experiences of sensory disturbance, her family, friends, and healthcare professionals often focus on her being cured from cancer, despite she has never felt so ill, why she no longer shares her concerns with them. Her lower confidence in her body is affecting her professional life, social life, and freedom. She can only work part time now. She has given up her dream of ‘walking the Camino’. She has become bodily slow and insecure; her ability to go by bike and her balance is affected negatively, and she needs to lean on tables or walls when she walks. Because of fatigue, she mostly feels comfortable being by her own although it also can lead to an increased attention on her discomfort. Mary manages her pain by wearing special shoes, walking with her dog, doing Yoga, sitting on her hands and other distracting activities such as watching television, knitting, doing crossword.

## Findings

**Figure 1.**
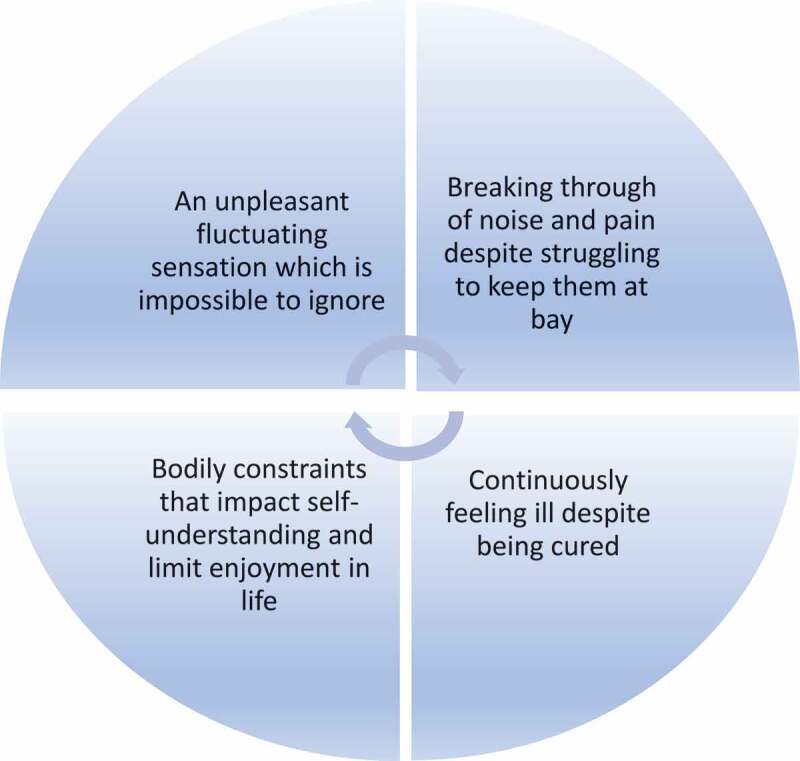
Essential qualities of the everyday experiences as a chemo sufferer.

Eight participants who displayed variation in age, gender, social and working status and cancer type contributed to the findings in this paper. Time since treatment cessation spanned from one year and three months to 11 years and six months (Table I). Despite this timespan, they all experienced an everyday life altered by sensory disturbances where their self-understanding and freedom to live their everyday life was changed and restricted. The explication of an essential meaning of how sensory disturbances caused by Oxaliplatin influenced the participants’ self-understanding and freedom to live their everyday life was intertwined with and structured by four constituents that illustrated what it meant to be sufferers after chemotherapy. The four constituents were: 1) An unpleasant fluctuating sensation which is impossible to ignore, 2) Breaking through of noise and pain despite struggling to keep them at bay, 3) Continuously feeling ill despite being cured, and 4) Bodily constraints that impact self-understanding and limit enjoyment of life (Figure 1).

### 1. An unpleasant fluctuating sensation which is impossible to ignore

The meaning of sensory disturbances revealed a shifting nature, one that fluctuated between the foreground and background of the participants’ bodily perception over the rhythm of the daily activity—often impossible to ignore. Jack, Dot and Mary were especially affected by sensory disturbances that were impossible to ignore at night. Mary described how they appeared without warning when she went to bed to rest and sleep

*Some nights and evenings, it is like flipping a switch when I go to bed (…) then it buzzes wildly in my hands and feet. It may well be a day where I have hardly noticed them, and then I go to bed and then they come. That is totally weird*.

Thus, at night Mary felt the sensory disturbances in hands and feet because at that time they became demanding. Being occupied during the day, Mary as an embodied self was directed against daily activities, and the sensory disturbances faded out and became unnoticed/silent in the background. However, solitude, being sedentary and inactive pushed the recognition of the disturbances. In these cases, being without any kinds of distraction, the disturbances were recognized as an overwhelming sensation appearing in the foreground that was impossible to ignore. It ushered Mary into a new way of being in the world where the attention was directed towards a weird demanding sensation impossible to escape—a symbol of a body suffering from late complications after chemotherapy. Karen added to Mary’s experience. She often perceived the sensory disturbances during the day, alongside activities, although they became more apparent when she turned her attention towards the body. Exploring her perception of her body in the interview, she said:

*It buzzes like that all the time, there is always a tingling in the feet up to here (…) and the hands. Now I can really feel it because we are talking about it. If I am busy with something, then I do not think about them, but often I register that it is there*.

Thus, the fluctuating nature of the sensory disturbances was not only related to inactivity. They also appeared when hands or feet were stimulated from outside in touching, as Jack said: *“When my hands are tranquil, I feel nothing but as soon as I touch something it appears”*. The unpleasant sensation also appeared evident when the participants walked on rough surfaces whether it was in the house or outside. Jean and Mary talked about the increased sensation when moving between different surfaces in the house and Dot told how she experienced an unpleasant feeling that intruded up through her body and left her with a feeling of walking disabilities when she walked on cobblestones outside her flat.

*If I walk in thin footwear on cobblestones, I feel everything up through my body. I am walking. However, I am not walking in a safe way because it feels unpleasant.*

Thus, the unpleasant sensations did not only display a fluctuating nature. They also appeared paradoxical because they appeared in the foreground in inactivity as well as in activity. Following this, the meaning of sensory disturbances was embedded in a bodily sensation of discomfort that extended in time and space, as Jean expressed it, “*even when they are not there, they are always there*”. Thus, the sensory disturbances as figures against the horizon—their everyday life activities, affected the participants’ bodily capacity—the “I can” and hence their freedom to act and to sleep and rest.

### 2. Breaking through of noise and pain despite struggling to keep them at bay

In the attempt to deliberately keep the sensory disturbances at bay—in the background, different actions and strategies were used e.g., biking, walking, gymnastics/yoga, knitting or concentrating on other activities like doing crossword puzzles or watching television. However, the distraction did not have a long-lasting effect. Being more or less “noisy” and being more or less painful, the meaning of sensory disturbances was an unpleasant sensation that broke through as soon as the activities ended. Although the participants tried to push the sensory disturbances into the background, they were no longer in control of their attention as the sensory disturbances interrupted their way of experiencing and interacting with the world despite the struggle to keep them at bay with different strategies. This was described by Mary who distinguished between sensory disturbances and joint pain.

*When I have a lot of sensory disturbances, I often sit on my hands. This induces joint pain which is easier to endure than the tingling, stinging and warm sensation in the hands (…) so if I cannot stand it, I just sit on my hands.*

In this case, Mary as an embodied self, was directed to an unpleasant body sensation with noise and pain. This forced her to choose between two forms of discomfort and to consciously keep her sensory disturbances at bay by an intellectual reflection to minimize what disturbed her the most compared to what she was capable of enduring. Sitting on her hands induced joint pain, which was easier to endure than the tingling, stinging and warm sensation. As opposed to Robert’s experience, which is described below, Mary’s action was not performed automatically. Robert’s action to prevent pain and strange feelings from breaking through was an unconscious one. An unpleasant sensation appeared when he sat on a chair without touching something, but when he touched his pants or the edge of a chair, the pain and odd feeling disappeared:

*The pain disappears when my hands touch the pants. I do it automatically. Also, if I pay a visit, quite unconsciously I grab hold of the chair or my pants, then the odd feeling is gone, it is just like nothing is left.*

The unconscious action signals that Robert had adapted to his bodily changes and his body scheme had reorganized into a new habit, so the unpleasant sensation was kept at bay. In this way, distracting actions aimed to force the unpleasant sensory disturbances into the background and support a pre-reflective being in the world—at least for a while. Thus, to prevent the sensory disturbances from breaking through with noise and pain and keep them at bay, the participants used distraction. In addition, paying attention to wearing special kinds of shoes or socks, being barefoot and using things like analgesic cream or menthol lotion figured in the participants’ toolboxes as means to suppress the disturbances. To get rid of the pain in her feet and fall asleep Jean said: *late in the evening I apply painkiller on both my feet. This can minimize my pain*. Dot also needed to keep the sensory disturbances at bay at night. She felt very disturbed if she went to bed barefoot and said:

*I have tried to go to bed without my cosy socks (socks that fit tightly on the feet), but it doesn’t work. When the temperature is high, I’ve tried to sleep without them, but then the disturbances are strongly present.*

Thus, the success in keeping the sensory disturbances at bay depended on how strongly they stayed in the foreground as gestalts impossible to avoid. Due to these conditions, the participants as unified body subjects seemed to be forced to understand that they needed to adapt to a new being where their ability to realize their bodily potential always had to take into account the presence of sensory disturbances and the struggle to keep them at bay. However, physical activity and other distractions seemed to contribute to minimizing the influence of the sensory disturbances in the performance of everyday life activities where the participants were given freedom to enjoy essential aspects of their life, though for a limited time.

### 3. Continuously feeling ill despite being cured

The meaning of suffering from sensory disturbances was also constituted as a divergence between being cured of a cancer disease and at the same time feeling ill due to the sensory disturbances. The chronic sensory disturbances prevented normal perception and became a constant reminder of having received treatment for a cancer disease, which was clearly articulated by Karen and Dot. According to Karen, the meaning of sensory disturbances was “connected to *an eternal reminder that I have had cancer. It would be nice not to have that reminder”*. Dot supported this quote by saying:

*I think it is because I’ve got chemo, I feel it that way. You are reminded of it EVERY single day and hope that it gets better, but unfortunately, this is not the case for me.*

The quotes illustrate a meaning of sensory disturbances that affected the participants’ self-perception as persons having had cancer and as sufferers after chemotherapy because they continuously felt ill. An everyday life with sensory disturbances became a life with an eternal reminder of a changed body perception where habits and everyday activities were reduced. Being hit by balance problems, decreased fine motor skills, sleep deprivation, lack of self-confidence and feeling exhausted were embedded in the participants’ bodies and affected the way they experienced the support from families and healthcare professionals. Being unable to sense the changes in the participants’ bodies, other people gave more attention to their being alive without recognizing what had been lost due to sensory disturbances. The suppression of their experiences when consulting the general practitioner for help or at control visits at the hospital appeared evident when Karen shared her experience of her control visit.

*The doctor said: ’If this is the price for being alive, it is surely okay?’ Yes, it is, but at a certain point of time in my life, I wish it would disappear’.*

Another quote that highlighted the illness experience despite being cured was expressed by Mary who said:

*The doctor said ‘Can’t you be happy that you are cured? ‘However, it is difficult to be happy that you are cured because I’ve never felt so damn ill.*

Thus, it seemed the participants had paid a price for being alive that was reasonable for physicians and others. However, some paid a price that influenced their ability to accomplish loved activities. For Carl, sensory disturbances in his feet prevented him from walking barefoot at the beach, and he, like Karen, articulated: “*I hope it disappears, so I no longer have to put up with these changes and am able just to walk barefoot again”*. Others had to refrain from certain leisure activities. Robert, who suffered from sensory disturbances in the hands, loved to work with radio mechanics. This is no longer easy for him..

*It is hard to pick up small screws in the radio. I can use a magnet to pick them up, but when I am going to assemble the items again, it is very difficult. But I try.*

Thus, the changes impacted on the participants’ quality of life in addition to the impact on basic living possibilities. When the sensory disturbances prevented Mary from sleeping at night, she paid the price of an overwhelming tiredness that forced her to change her full-time employment to working part-time to ensure enough time to rest. Others—John and Carl—were forced to take early retirement because they were no longer able to perform fine motor skills. In this way, the participants’ bodily constraints and the fact that they felt ill despite being cured affected their freedom to return to their former everyday life as working people, a condition that could make them feel disabled and affect their self-understanding.

In addition, lacking understanding from those around them regarding the discrepancy between being cured and feeling ill, it became difficult for the participants to share their concerns with their families, friends, and healthcare professionals. Thus, the price for being alive was not only an everyday life with the perception of a changed body. The sensory disturbances affected their being-in-the-world, which included their everyday life contexts such as their relationships with other people. Furthermore, the participants’ experiences were contrasted with the view of a culture that focused more on their survival and not the restoration to a healthy body. What others called the price of being alive, induced in the participants a struggle to adapt to bodily changes and a new self-understanding as sufferers after chemotherapy while still hoping the sensory disturbances would fade away and disappear. Thus, the participants’ existence was disrupted by a present body with serious sensory disturbances, a deviance from the habitual body and the ability to act pre-reflective and move in time and space easily.

### 4. Bodily constraints that impact self-understanding and limit enjoyment in life

The last constituent that forms the essential meaning of sensory disturbances is their impact on self-understanding and the ability to enjoy oneself. To keep up one’s former self-understanding and to prevent being exposed as a sufferer after chemotherapy in public, as incapable of doing certain things in their everyday life, the participants described how they avoided performing activities they used to do, keeping themselves safe but limited. John loved to take a run in the forest but now he was afraid of falling due to the changed sensation in his feet.

*I don’t think it is a good idea to run in the forest anymore. I haven’t done that because there are a lot of up and down hills and different path levels when you run there. I think it would be too dangerous.*

The quote illustrated how John delimited himself from a physical activity he used to find joy for fear of being injured. The withdrawal from enjoyable activities and the urge to recognize bodily constraints appeared evident in several of the participants’ descriptions. Robert enjoyed playing the concertina and had been a member of a concertina band for many years, but the sensory disturbances in his hands made it difficult for him to hit the keys and feel confident in playing new melodies.

*I have declined to play together with the others next Sunday at a concertina event. It was difficult for me, because we were going to play a new melody and I felt unable … not good enough. Then I knew, now this old man can’t keep up with the others.*

The consequences of suffering after chemotherapy changed Robert’s self-understanding from one of being able to enjoy himself and other people, to an old man that hid his weakness by withdrawing from the group not to expose himself as one that played the instrument wrongly and disturbed the harmony. Mary, who used to be active and keep in contact with her two sisters and other family members no longer had the energy to phone them as usual. Consequently, she felt more alone although *“it is not because we don’t want to talk with one another, but I used to take the initiative, which I no longer have the strength to”*. However, to keep up with a kind of social life, Mary compensated for her missing strength by organizing and structuring when she invited guests.

*I agreed to making Christmas lunch for 17 people. (…). I delegated the two things that took the longest time (…). I bought herrings, shrimps, and eggs etc. Then it seemed like I was still able to host a Christmas event. Then they say: “Well, you’ve even baked bread rolls’. And they don’t know they had been in the freezer for three weeks*

Although Mary had given up her dream of “walking the Camino de Santiago”, she struggled to maintain being a sister and a family member who “**can**” in her attempt to preserve her social life and relationship with others. Thus, it seemed that it was very urgent for the participants to outwardly show that they were still the same person even though they experienced themselves as limited sufferers after chemotherapy. In order not to dwell too much on these losses, and other people’s reactions, they more and less consciously chose to hide these weaknesses in their attempt to escape a part of their reality. However, a new latent background, in the sense of an inability to keep up with life as it was, kept jumping into the foreground of the participants’ lives and impacted profoundly their self-understanding. Furthermore, being confronted by a lack of understanding from the people around them may lead to loneliness and prevent the participants from receiving appropriate care.

Summarizing the findings

The four constituents illustrated what appeared stable within the phenomenon, which was “an everyday life as a sufferer after chemotherapy”. The constituents were bound together by sensations that fluctuated between the foreground and background of the participants’ bodily perceptions as sufferers after chemotherapy. Appearing in the foreground as unpleasant bodily sensations, the sensory disturbances became noisy and demanding and impossible to ignore. With the breaking through of noise and pain the participants struggled to deliberately keep the sensory disturbances at bay by using different actions and strategies. However, everyday life was permeated with a sense of being ill despite being cured of the cancer disease. Bodily constraints forced the participants to adapt to their present bodily state and refrain from activities that earlier contributed joy and the maintenance of relationships to their lives. Thus, their former self-understanding and freedom to live their everyday lives as before was replaced by a new way of being-in-the-world, marked by a wounded self-understanding which included grieving from becoming a sufferer after chemotherapy, and which urged them to hide their weaknesses (Figure 1).

## Discussion

This study aimed to explore the essential meaning on how sensory disturbances caused by Oxaliplatin influence the participants’ self-understanding and freedom to live their everyday life. Four constituents formed the essential meaning, which during the analysis emerged as a requirement for the participants to adapt to a new self-understanding as sufferers after chemotherapy, a condition that limited their possibility to perform everyday life activities. Appearing on a fluctuating basis which was impossible to ignore, the sensory disturbances broke through as unpleasant sensations with noise and pain irrespective of resting or being active. The influence of sensory disturbances became essential while falling asleep, fine motor skills etc. were affected. The unpleasant feelings of tingling, pinpricking, and pain in hands and feet were occasionally diminished by using analgesics, different forms of lotion, and socks or by inducing a preferable painful sensation instead when sitting on hands as self-care actions. Using different self-care and pharmacological strategies to modify the intensity of sensory disturbances are in accordance with the results of the study by Bakitas (2007). Still, when Bakitas (2007) found the participants adapted to the changes using cognitive processes such as minimizing, denying, or ignoring symptoms, the participants in our study demonstrated how they used distraction deliberately to keep the sensory disturbances at bay. Distraction like thinking about something else as a coping strategy was found useful to minimize distressing bodily symptoms among women treated for breast cancer (Browall et al., 2016). In addition to thinking about something else, distracting actions to minimize concurrent symptoms could be keeping busy, reading, doing crosswords, watching TV/movies, and isolating oneself from others (Dong et al., 2016).

Distracting actions in our study appeared as an attempt to keep the mind busy and being occupied with activities that required bodily activity. However, due to the fluctuating nature of the sensory disturbances, they also appeared paradoxical as some of the distracting activities also contributed to recognizing the sensory disturbances. It was this recognition that forced the participants to adapt to a new self-understanding as sufferers after chemotherapy—a self-understanding that was further formed by the fact that sensory disturbances were understood by participants, families and healthcare professionals as paying the price of being alive. Despite their being cured of their cancer disease, the participants experienced themselves as an unhealthy body subject, revealed in Mary’s statement of never having been feeling “so damn ill”.

Key concepts like health, sickness, illness, and disease are being diversely and ambiguously used (Kottow, 2017), and according to Farre and Rapley (2017), the term disease is typically based on a biomedical, physiological understanding of the body whereas the term illness encompasses an objective as well as a subjective experience called ill-health (Farre & Rapley, 2017). Our study revealed that despite being free from cancer disease, long-term sensory disturbances disrupted everyday life. In addition to being perceived as the price of being alive, the freedom to live life as usual was affected, which indicates that the participant recognized their condition as ill health.

The influence of symptoms on everyday life activities corresponds with findings in other studies. In the study of Tanya and Arms **(2019)**, sensory disturbances made the participants feel disabled and socially isolated. These consequences are found to induce frustration, anger and depression (Bakitas, 2007; Tofthagen, 2010) and other emotional challenges like fear and helplessness (Kanda et al., 2017). To add to these serious consequences, Thong et al. (2018) found depression and significantly lower quality of life among a significant proportion of cancer survivors that still consider themselves to be patients five to 15 years after being diagnosed with cancer (Thong et al., 2018). Thus, it seems that sensory disturbances contribute to constraints regarding the transition to an ordinary everyday life and maintenance of one’s former self-understanding. An interesting aspect was the interpretation of the self-understanding as a sufferer after chemotherapy found in our study. Taking this perspective, the participants did not identify themselves as cancer survivors although other studies point at that label after cancer treatment (Cheung & Delfabbro, 2016; Khan et al., 2012). In a study of Khan et al. (2012) some understood “cancer survivor” as having survived cancer. For others, the term meant a higher risk of death and was a label and an identity they did not want to adopt (Khan et al., 2012). According to Cheung and Delfabbro (2016), the identification with being a cancer survivor depended on the experience of symptoms. When there were no symptoms there was a tendency to identify oneself as a survivor but when suffering from symptoms, it became more likely to perceive oneself as a patient (Cheung & Delfabbro, 2016). In our study, the experience of everlasting sensory disturbances indicates a feeling of misery and a continuous struggle for returning to the habitual body, which may indicate a self-understanding as still feeling like being a patient. Chambers et al. (2012) and Cheung and Delfabbro (2016) found that people who adopted the identity of being a cancer survivor reported more positive outcomes after cancer, higher quality of life and a better mental being (Chambers et al., 2012; Cheung & Delfabbro, 2016). Thus, it is worth considering whether survivors that identify themselves as sufferers after chemotherapy with a wounded self-understanding are exposed to lower quality of life and depression.

A healthy body is characterized by flexible and spontaneous movements and an unhealthy body by fixed, non-spontaneous movements that require intellectual reflection (Moya, 2014). Feeling that they suffer ill-health, the participants may be affected in their pre-conscious system of bodily movements and situational spatiality—the “body scheme” or their habitual way of action (Merleau-Ponty, 2014). Consequently, the sensory disturbances may affect the way the embodied self becomes a specific embodied person that expresses itself in a specific stylistic way different from other people (Forlé, 2019). The participants in our study were affected in their personal style of action and withdrawal from social contacts and they avoided activities that would expose them as sick or disabled in accordance with other studies (Drury et al., 2021; Tanay & Armes, 2019). Thus, they minimized the risk of being compared with other persons and seeing oneself in the eyes of others, which according to Merleau-Ponty (2014) is a price to pay for being in the world (Merleau-Ponty, 2014). Following this, the disruption of everyday life was extensive and involved physio- and psychosocial aspects and affected the participants as unified body-subjects formed by bodily interactions with the world, culture and environment (Merleau-PontyMerleau-Ponty, 2004, 2014). As the participants experienced that people in their surroundings did not acknowledge the excessive influence of sensory disturbances on their everyday life, they were left alone to manage their symptoms when they paid the price for being alive, according to the studies of Drury et al. (2021) and Tanay and Armes (2019). Thus, our findings suggested, the participants also were affected by a cultural expectation of understanding of being cured and free of cancer is the same as having completely recovered. Consequently, dealing with the disruptive effect of ill health requires a clinical attention to all domains of human life, as the boundaries between health and illness, between well(ness) and sick(ness), are diffused by cultural, social, and psychological considerations (Farre & Rapley, 2017).

The strong emphasis on being a sufferer after chemotherapy showed how deep an impact the sensory disturbances had on self-understanding. According to Forlé (2019), the unique personality emerges not only through narrative practices but in an expressive and pre-reflective way that is connected to the experience of oneself and one’s relations to other—an embodied expressive style of the “I can” (Forlé, 2019). The participants in our study showed how they refrained from actions they used to enjoy in their attempt to maintain their former self-understanding. They stopped playing their instrument, running in the forest and diminished their social contact. The body as an organ of the will performing free and spontaneous movements intertwined in a world of practical meanings and possibilities for actions could no longer be trusted. Thus, they were affected in their what Forlé (Forlé, 2019) calls their former pre-reflective and primordial experience and sense of agency—the “I do” and the “I can”, which is why the changes in the participants’ personal style of behaviour found in our study seemed to be a double-edged sword. In the attempt to hide their weaknesses and maintain their life as they knew it, the participants’ actions—withdrawal from former activities, showed how they were no longer the person they used to be, and illuminated a wounded self-understanding due to being thrown into a new being-in the-world as sufferers after chemotherapy.

### Strengths and limitations

Measures to enhance the scientific rigour and trustworthiness of the study were obtained through the study process as we followed the methodological process guided by existential phenomenology and reflective lifeworld research (Dahlberg & Dahlberg, 2019; Merleau-Ponty, 2014). The sample in phenomenological studies must consist of 10 or fewer persons, and they may have shared a common experience concerning the phenomenon under study despite different demographic characteristics (Polit & Beck, 2012). Thus, the sample size was relevant to the study design. Some interviews were short even though the interview was based on a structure for phenomenological interviewing (Bevan, 2014). However, including participants after CRC of different gender, ages, timespan after treatment completion and working conditions contributed to a variety of experiences of living with sensory disturbances. Still, the enrolment of participants via social media or by nurses in the outpatient unit may be a limitation as only those who had access to the platform or visited the outpatient clinic were invited.

The strength of the study and its trustworthiness was enhanced as all researchers focused on the lived experience of the participants and its meaning. Furthermore, all researchers contributed to the analysis with their different professional perspectives. This slowed down the analysis in order to pose questions to the text and ourselves as recommended for a phenomenological exploration as well as the opportunity to obtain intercoder agreement (Dahlberg et al., 2008). In this process, the truth value was supported as the researchers’ prejudices were challenged to ensure the findings represented the participants’ lived experience. Finally, the exemplarity of the findings (Feilberg et al., 2018) makes those in this study relevant not only to patients diagnosed with CRC receiving oxaliplatin. They are also relevant for other patient groups receiving oxaliplatin or similar nerve-damaging drugs as well as to all contexts where patients suffer from the loss of former capabilities as well as sensory disturbances in its widest sense.

## Conclusion

Using an existential-phenomenological approach to exploring everyday life with sensory disturbances added a new and increased understanding of how sensory disturbances affect self-understanding and freedom to conduct everyday life activities among survivors after CRC. Firstly, to maintain an everyday life, the participants used distraction to keep the sensory disturbances at bay. However, due to the paradoxical and shifting nature of the disturbances, they were forced to adapt to a new way of being-in-the-world despite being cured of their cancer disease. Secondly, the participants in this study, their families and healthcare professionals could understand these consequences as a necessary price to pay to be alive. Nevertheless, the essential meaning of our study highlights the true price of being alive—that the participants’ everyday style of experience and life has been irrevocably marked as sufferers after chemotherapy revealed as an ill health condition. This new life affected the participants’ interrelatedness with the world, culture and environment. The embodied sensory disturbances revealed as a figure in the background of a former body that used to be free to realize its potential—the “I can”, but which was now lost. Finally, to hide their weaknesses and inability to accomplish everyday life activities like before, the participants diminished their former activities and social contacts. Although this action minimized the risk of seeing oneself in the eyes of others, this had the potential to be a two-edged sword as this action exposed them as chemo-sufferers and underscored their perception of still being a patient.

## Relevance for clinical practice

This study provides essential knowledge about everyday life with chronic sensory disturbances after treatment with oxaliplatin and sheds light on the often implicit and pre-reflective structures and habits that characterizes these participants’ lives as sufferers after chemotherapy. Thus, taking an existential-phenomenological approach contributes to exploring an underexposed topic, adds to the sparse literature, points at the need for further research to support a group of people that needs attention in the healthcare sector—at hospitals as well as in general practices. Labelling the sensory disturbances as a necessary “price” paid for survival points at a risk of neglecting the late complications that these participants live with. Therefore, there is a need for increased attention on this topic. In addition to the impact of sensory disturbances among survivors treated for CRC, one-third to one-half of cancer survivors experience existential distress and fears due to loss of control, identity and insecurity about the future (Vehling & Philipp, 2018). These facts combined with the risk that the sensory disturbances could be a lifelong companion with no treatment adds to the complexity of caring for these survivors. To provide caring support to people suffering from sensory disturbances instead of leaving them alone with their symptoms, knowledge sharing about the serious impact of sensory disturbances found in several studies (Lim et al., 2021; Pedersen, Jensen, Yilmaz, Mørch, Feilberg et al., 2021; Tanay & Armes, 2019; Tanay et al., 2017) could be relevant in public healthcare and across professionals in hospitals, communities and general practitioners. Additionally, a tangible action like establishing existential conversation could contribute to sharing grief over cancer-related losses (Vehling & Philipp, 2018). Using this approach may support hope and engagement for survivors after cancer and thus also for survivors suffering from sensory disturbances. However, there persist gaps in the literature on how to support survivors after cancer. Greenblatt and Lee (2018) recommend follow up studies of CRC patients with both quantitative and qualitative methodology. In addition, interventions to promote early detection of CIPN, including effective reporting and assessment of it are warranted to minimize the risk of developing chronic sensory disturbances and subsequent effects on quality of life.
